# The Host Protein Aquaporin-9 is Required for Efficient *Plasmodium falciparum* Sporozoite Entry into Human Hepatocytes

**DOI:** 10.3389/fcimb.2021.704662

**Published:** 2021-06-29

**Authors:** Nadia Amanzougaghene, Shahin Tajeri, Samir Yalaoui, Audrey Lorthiois, Valérie Soulard, Audrey Gego, Armelle Rametti, Véronica Risco-Castillo, Alicia Moreno, Maurel Tefit, Geert-Jan van Gemert, Robert W. Sauerwein, Jean-Christophe Vaillant, Philippe Ravassard, Jean-Louis Pérignon, Patrick Froissard, Dominique Mazier, Jean-François Franetich

**Affiliations:** ^1^ Sorbonne Université, INSERM, CNRS, Centre d’Immunologie et des Maladies Infectieuses, CIMI-Paris, Paris, France; ^2^ Université Pierre et Marie Curie-Paris 6, UMR S945, Paris, France; ^3^ INSERM, U945, Paris, France; ^4^ EnvA, UPEC, Anses, Dynamyc research group EA 7380, Maisons-Alfort, France; ^5^ Department of Medical Microbiology, Radboud University Nijmegen Medical Centre, MMB-NCMLS, Nijmegen, Netherlands; ^6^ AP-HP, Service de Chirurgie Digestive, Hépato-Bilio-Pancréatique et Transplantation Hépatique, Centre Hospitalo-Universitaire Pitié-Salpêtrière, Paris, France; ^7^ CR-ICM - LGN CNRS UMR-7991, IFR des Neurosciences, Groupe Hospitalier Pitié-Salpêtrière, Paris, France

**Keywords:** *Plasmodium falciparum*, sporozoites, liver stage, hepatocytes, Aquaporin-9, CD81

## Abstract

Hepatocyte invasion by *Plasmodium* sporozoites represents a promising target for innovative antimalarial therapy, but the molecular events mediating this process are still largely uncharacterized. We previously showed that *Plasmodium falciparum* sporozoite entry into hepatocytes strictly requires CD81. However, CD81-overexpressing human hepatoma cells remain refractory to *P. falciparum* infection, suggesting the existence of additional host factors necessary for sporozoite entry. Here, through differential transcriptomic analysis of human hepatocytes and hepatoma HepG2-CD81 cells, the transmembrane protein Aquaporin-9 (*AQP9*) was found to be among the most downregulated genes in hepatoma cells. RNA silencing showed that sporozoite invasion of hepatocytes requires AQP9 expression. AQP9 overexpression in hepatocytes increased their permissiveness to *P. falciparum*. Moreover, chemical disruption with the AQP9 inhibitor phloretin markedly inhibited hepatocyte infection. Our findings identify AQP9 as a novel host factor required for *P. falciparum* sporozoite hepatocyte-entry and indicate that AQP9 could be a potential therapeutic target.

## Introduction

Of the protozoan parasites of humans, *Plasmodium falciparum* is the deadliest, accounting for an estimated 228 million cases of malaria and causing 405 000 deaths in 2018 (WHO | World malaria report 2018). After being inoculated in the skin of a host during the bite of an infected *Anopheles* mosquito, the sporozoite form of the parasite is quickly conveyed by the blood to the liver. Once there, it is sequestered *via* interactions with heparan sulphate proteoglycans (HSPGs), crosses the endothelial barrier, and then migrates through several parenchymal cells before invading a hepatocyte by forming a membrane-bound compartment called the parasitophorous vacuole membrane (PVM), where it can develop and multiply to form merozoites that initiate the pathogenic erythrocytic cycle (Mota et al., 2001).

Sporozoite entry into hepatocytes is a highly complex process that involves coordinated interaction of sporozoite and host factors for initiation of infection, which may offer a multitude of promising therapeutic targets (Dundas et al., 2019). Currently, two major host cell surface proteins are well known to be involved in sporozoite invasion, the tetraspanin CD81 and the scavenger receptor class B type1 (SR-BI) (Silvie et al., 2003; Rodrigues et al., 2008; Yalaoui et al., 2008a), but how they interact with specific parasite ligands remains largely unknown (Silvie et al., 2007). Moreover, the molecular events involved in sporozoite invasion are further complicated because they depend on the plasmodial species and the host cell type (Risco-Castillo et al., 2014). Indeed, CD81 is essential for *P. falciparum* and *P. yoelii* sporozoite invasion (Silvie et al., 2003) but is not required by *P. vivax*, which depends only on SR-BI. On the other hand, SR-BI is not required for *P. falciparum and P. yoelii* infections (Manzoni et al., 2017). However, *P. berghei* sporozoites can enter cells alternatively *via* either CD81 or SR-BI (Manzoni et al., 2017). The choice of route by which sporozoites invade hepatocytes has been shown to be determined by the parasite 6-cysteine domain protein P36 in a species-specific manner (Manzoni et al., 2017). In addition, SR-BI has been found to be essential for subsequent parasite development inside hepatocytes but not CD81 (Silvie et al., 2003; Rodrigues et al., 2008). Most recently, another host factor, aquaporin 3 (AQP3), which is a water and glycerol channel belonging to the aquaglyceroporin subfamily, has been implicated in the maturation of parasites after invasion (Posfai et al., 2018; Posfai et al., 2020).

Previous studies have shown that the human hepatocarcinoma HepG2 cells express SR-BI but not CD81, and as a consequence, they support infection by *P. berghei* but not by *P. falciparum* or *P. yoelii* sporozoites (Silvie et al., 2006b; Manzoni et al., 2017). Interestingly, ectopic expression of CD81 in HepG2 cells is sufficient to confer susceptibility to *P. yoelii* but not to *P. falciparum* sporozoites (Silvie et al., 2006b), implying the existence of additional host factors that are critical for *P. falciparum* infection.

The aim of this study was to identify host proteins required for successful infection of human hepatocytes by *P. falciparum* sporozoites. The approach adopted was that of a differential transcriptome analysis of naturally permissive human hepatocytes *versus* non-permissive hepatoma HepG2-CD81 cells. Among the most downregulated genes in the non-permissive cells, we focused on that coding for aquaporin-9 (AQP9), a water and glycerol channel previously shown to be involved in mice susceptibility to *P. berghei* blood stage infection (Liu et al., 2007). The contribution of AQP9 to the *Plasmodium* liver stage of *P. falciparum*, *P. yoelii* and *P. berghei* was investigated using primary human hepatocytes, human hepatoma cells and primary murine hepatocytes.

## Materials and Methods

### Ethics Statement

Human hepatocytes were isolated from liver segments taken after oral informed consent was obtained from adult patients undergoing partial hepatectomy as part of their medical treatment (Service de Chirurgie Digestive, Hépato-Bilio-Pancréatique et Transplantation Hépatique, Hôpital Pitié-Salpêtrière, Paris, France). The collection and use of this material for the purposes of the study presented here were undertaken in accordance with French national ethical guidelines under Article L. 1121-1 of the ‘‘Code de la Santé Publique’’. Given that the tissue samples were classed as surgical waste, they were used anonymously. Moreover, because they were not in any way genetically manipulated, article L. 1211-2 stipulates that their use for research purposes is allowed provided that the patient does not express any opposition to this to the surgeon prior to surgery after being informed of the nature of the research in which they might be potentially employed. The collection and use of this material were furthermore approved by the Institutional Review Board (Comité de Protection des Personnes) of the Centre Hospitalo-Universitaire Pitié-Salpêtrière, Assistance Publique-Hôpitaux de Paris, France. All animal care and procedures were carried out in strict accordance with the recommendations of the Guide for the Care and Use of Laboratory Animals of the European Union “European directive 86/609/EEC”. The protocol was approved by the Ethics Committee for Animal Experiments of University Pierre et Marie Curie, Paris 6, France (Permit Number: 75-1087).

### RNA Isolation, Microarray Protocol and Differential Analysis of Transcriptomes

Three sets of human primary hepatocytes **(** **Supplementary Table 1** **)** were cultured for one week in a differentiation maintenance medium (complete medium for hepatocyte culture supplemented with 2% DMSO) (Isom et al., 1985). Hepatoma-derived HepG2-CD81 cells (Silvie et al., 2006b) from three different cultures were grown to subconfluence in 75 cm² flasks. Cells were lysed with TRIzol (Invitrogen, Life Technologies), and RNA was purified according to the manufacturer’s instructions. RNA was equally purified from 1 g of human liver for use as a reference in all the microarray hybridizations. RNA concentration and purity were assessed using a NanoDropND-1000 Spectrophotometer (Thermo Scientific) and the Agilent 2100 Bioanalyser.

Transcriptomes of *P. falciparum* permissive human hepatocytes (HHs) and refractory HepG2-CD81 hepatoma cells (HCs) were analysed using a human 1A oligoarray kit (pangenomic human 22K DNA microarrays from Agilent Technologies, Les Ulis, France). Cyanine-labelled cDNA probes were synthesized from 20 μg of HH, HC, or total liver RNA using a CyScribe Post-Labelling kit (GE Healthcare Life Sciences, Vélizy, France). cDNA probes synthesized from cell RNAs were labelled with cyanine 3 (Cy3), while probes synthesized from the liver reference RNA were labelled with cyanine 5 (Cy5). Cy3- and Cy5-labelled probes were then co-hybridized to spots on the same microarray. Signals were extracted using a Genepix 4000B scanner (Axon Instruments) and Genepix Pro 6.0 software and normalized with VARAN software (http://www.bionet.espci.fr/varan/), allowing calculation of the HH/HC intensity ratio for each targeted gene spotted on the microarray as the ratio of (HH/reference intensity ratio) *versus* (HC/reference intensity ratio). All microarray procedures were performed in accordance with the manufacturer’s instructions. The CD81 synthetic oligonucleotide probe used here is complementary to the endogenous 5’-untranslated region of wild type *CD81* but not to the ectopic *CD81* expressed by the HepG2-CD81 cell line, therefore the probe recognizes only endogenous *CD81* mRNA.

### Hepatocytes, Hepatoma Cells and Cell Culture

Primary human hepatocytes were isolated from healthy parts of the liver fragments as previously described (Yalaoui et al., 2008a). Primary mouse hepatocytes were isolated as previously described (Yalaoui et al., 2008a) from *AQP9^-/-^* mice backcrossed onto a C57BL/6J background and their *AQP9^+/+^* littermate controls aged 7 to 10 weeks and gender matched (Rojek et al., 2007). These mice were kindly provided by Pr. S. Nielsen (Aarhus University, Aarhus, Denmark) and Pr. M. Amiry-Moghaddam (University of Oslo, Oslo, Norway). Human and mouse hepatocytes were seeded at a density of 8 × 10^4^ cells and 4× 10^4^ cells, respectively, in 96-well microplates precoated with rat tail collagen I (Becton Dickinson) and cultured at 37°C in 5% CO_2_ in William’s E medium (Gibco, Life Technologies, Saint Aubin, France) supplemented with 2% penicillin-streptomycin, 1% sodium pyruvate, 1% L-glutamine, 1% insulin-transferrin-selenium, 1% non-essential amino acids (solutions for cell culture, Gibco) and 10% foetal bovine serum (FBS, Gibco). For human hepatocyte, the culture medium was also supplemented with 10^–7^ M dexamethasone (Sigma) and 2% DMSO. HepG2-CD81 (Bartosch et al., 2003; Silvie et al., 2006b) human hepatoma cells were cultured in DMEM (Gibco) supplemented with 10% FBS, 1% glutamine and 1% penicillin-streptomycin (Gibco). HepaRG human hepatoma cells, a gift from Pr. C. Guillouzo (INSERM, Rennes, France), were cultured in William’s E medium (Gibco) supplemented with 1% penicillin-streptomycin, 1% L-glutamine, insulin (5 µg/ml), 5 × 10^-5^ M hydrocortisone and 10% FBS (Thermo Scientific, Villebon-sur-Yvette, France). Mouse and human hepatocytes as well as human hepatoma cells were cultured for at least 24 hours before further studies.

### *Plasmodium* Parasites and *In Vitro* Infection

*P. yoelii* (265BY strain), *P. berghei* (PbGFPCON, ANKA strain) (Franke-Fayard et al., 2004) and *P. falciparum* (NF54 strain) sporozoites were obtained by dissection of salivary glands from infected *Anopheles stephensi* mosquitoes bred and infected in the insectary facilities of UMRS 945 (Paris, France) for rodent *Plasmodium-*infected mosquitoes and the Department of Medical Microbiology, Radboud University (Nijmegen, Holland) for *P. falciparum*-infected mosquitoes. Infection assays were performed in 96-well plates. Cells were inoculated with 3 x 10^4^ P*. falciparum* or 2 x 10^4^ P*. berghei* or *P. yoelii Plasmodium* sporozoites per well and then centrifuged for 10 min at 2000 rpm to allow fast parasite sedimentation onto the target cells. After 3 hours at 37°C/5% CO_2_, thus allowing sporozoite penetration into hepatocytes, cultures were washed and further incubated in fresh medium for 2 or 4 days (for *P. yoelii* and *P. berghei* or *P. falciparum*, respectively) until quantification of EEFs or directly fixed in PFA for quantification of parasite entry.

### Gene Knockdown Using Small Interfering RNAs

For gene knockdown, we used small double-stranded RNA oligonucleotides (siRNAs) targeting human *CD81* (sihCD81; 5’- GCA CCA AGT GCA TCA AGT A -3’), human *AQP3* (AQP3_5 siRNA from Qiagen HP GenomeWide), human *AQP9* (sihAQP9^1^; 5’-CTG CTG ATC GTG GGA GAA A-3’ and sihAQP9s^2 to 5^ corresponding respectively to siRNAs from Qiagen HP GenomeWide - AQP9_1 HP siRNA; AQP9_2 HP siRNA; AQP9_4 HP siRNA; AQP9_5 HP siRNA), and hCD92 (sihCD92; 5’- AAG GCA AGA ACT GAA AAC T -3’), used as a negative control. Primary hepatocytes were transfected with 30 nM siRNA in 96-well microplates using Lipofectamine RNAi MAX (Invitrogen) according to the manufacturer’s recommendations. HepaRG cells (5 × 10^6^ cells in 400 µl of RPMI) were transfected with 200 pmol of siRNA *via* electroporation (300 V, 500 µF) (Silvie et al., 2006a) using a Gene Pulser apparatus (Bio-Rad,Ivry, France). siRNA-transfected hepatoma cells or hepatocytes were then cultured for 48 hours before mRNA expression analysis or for 72 hours before protein expression or sporozoite infection efficiency analysis.

### Lentiviral Vector Constructs and Cell Transduction

The lentiviral constructs pTRIP-CMV-hAQP9 ΔU3 and pTRIP-CMV-hCD81 ΔU3 were generated *via* gateway recombination cloning (Invitrogen). Briefly, hAQP9 and hCD81 coding sequences were PCR amplified and cloned into a pENTR/D/TOPO plasmid (Invitrogen) to generate entry clones. LR clonase II recombination was performed using the entry clones and the pTRIP-CMV-rfa Gateway ΔU3 destination vector as described previously (Russ et al., 2008). The pTRIP-CMV-GFP ΔU3 vector (Castaing et al., 2005) was used as a control. Lentiviral vector stocks were produced by transient transfection of 293T cells with the p8.91 encapsidation plasmid (Zufferey et al., 1997), the VSV glycoprotein-G encoding pHCMV-G plasmid (Yee et al., 1994), and the pTripΔU3 lentiviral vector as previously described (Zennou et al., 2000). Supernatants were treated with DNAse I (Roche Diagnostics) prior to ultracentrifugation, and the resulting pellet was resuspended in PBS, separated into aliquots and frozen at 80°C until use. Primary human hepatocytes and hepatoma cells were transduced through addition of a 1:1000 concentrated lentiviral preparation. After an incubation period of three hours, the cells were washed. Three days later, the cultures were processed for protein expression analysis as described in western blotting procedures or infected with sporozoites and cultured for four days until exo-erythrocytic forms (EEFs) quantification to evaluate hepatocyte permissiveness to *Plasmodium* infection.

### Quantification of hAQP9 and hCD81 Transcripts *via* Real-Time PCR

RNA extractions were performed with a PureLink RNA Mini Kit (Ambion, Life Technologies) according to the manufacturer’s recommendations. DNase treatment (PureLink DNase Set; Ambion, Life Technologies) was performed during the extraction. The quality and purity of RNAs were assessed with the Agilent 2100 Bioanalyser. Reverse transcription was realized with a SuperScript VILO cDNA Synthesis Kit (Invitrogen) according to the manufacturer’s instructions, using 1 µg of RNA in a 20 µL final volume and duplicates for all samples. Quantitative real-time PCRs (qPCRs) were carried out using the SYBR Green method (SYBR GreenER qPCR SuperMix Universal, Invitrogen) with a MX3005P qPCR System (Agilent Technologies). The volume of cDNA used was equivalent to 100 ng of RNA per reaction, and the primers were used at a final concentration of 200 nM. Primer pairs based on the cDNA sequence were specifically designed for h*AQP9* (5’-ttgcaacatacccagctccgtatc-3’ and 5’-accaaagggcccactacaggaat-3’) and for h*CD81* (5’-.tcatcctgtttgcctgtgaggt-3’ and 5’-tgaggtggtcaaagcagtcagt-3’). All samples were run in duplicate with the two different cDNAs synthesized, and gene expression data were normalized according to the level of *TBP* (TATA box-binding protein) (Ceelen et al., 2011).

### Protein Expression Analysis *via* Western Blotting and Cell Immunofluorescence

Primary human hepatocytes were lysed at 4°C for 30 minutes in 30 mM Tris (pH 7.4), 150 mM NaCl, 0.02% NaN_3_, protease inhibitors (PMSF, INH) and 1% Triton X-100. *CD81* and *AQP9* expression was analysed *via* western blotting using a mouse anti-hCD81 mAb (1:1000) and a mouse anti-m/hAQP9 mAb (G3, Santa Cruz) (1:100), respectively, followed by a goat anti-mouse Alexa Fluor 680 conjugate (Molecular Probes, Life Technologies) (1:15000). Monoclonal mouse anti-tubulin (Abcam) (1:5000) was used as a standard. Data were acquired and quantified with an Odyssey Infrared Imaging System (LI-COR Biosciences, Lincoln, NE).

Quantification of h*CD81* and *AQP9* expression at the surface of human hepatocytes was performed as previously described for murine hepatocytes (Yalaoui et al., 2008a). Cells were washed with PBS, and immunolabelled for human AQP9 using a goat anti-hAQP9 (C18, Santa Cruz) antibody and a donkey anti-goat IgG-Alexa 488 conjugate antibody (Molecular Probes). CD81 was labelled with a mouse anti-hCD81 antibody (TS81, provided by Dr. E. Rubinstein, INSERM, Villejuif, France) and revealed with donkey anti-mouse IgG-Alexa 568 (Molecular Probes). Cell density was evaluated with diamidino-phenyl-indole (DAPI, Sigma) and used to normalize *CD81* expression. Fluorescence intensity was acquired with a Flexstation Universal Microplate Reader (Molecular Devices, St Grégoire, France).

### Quantification of Parasite Infection or Invasion *via* Immunofluorescence

After fixation of cultures with cold methanol, EEFs were identified using anti-HSP70 serum, prepared from mice immunized with a recombinant HSP70 protein (a gift from Pr. D. Mattei, Institut Pasteur, Paris, France), stained with an Alexa 488-conjugated or Alexa Fluor 680-conjugated goat anti-mouse immunoglobulin (Molecular Probes) and counted under a fluorescence microscope or using the Odyssey Infrared Imaging System as described previously (Gego et al., 2006). Nuclei were stained with 1 μg/ml DAPI. Quantification of parasite entry was carried out using double immunostaining with the appropriate mouse monoclonal anti-CSP antibody for *P. falciparum* CSP*^P. falciparum^* and *P. yoelii (*CSP*^P. yoelii^*) (Dr. Y. Charoenvit, Naval Medical Research Center; Silver Spring, MD), or a TRAP antibody for *P. berghei* (Dr. A. Crisanti, Imperial College, London, UK), thus allowing discrimination of intracellular and extracellular sporozoites, as previously described (Rénia et al., 1988; Silvie et al., 2002).

### Sporozoite Cell Traversal Assay

Hepatocytic cell traversal was analysed using a dextran incorporation FACS assay (Prudêncio et al., 2008). Immediately after electroporation in the presence of siAQP9, siCD81, or siCD92, HepaRG cells were plated and cultivated in a 25 cm^2^ culture flask at 37°C/5% CO_2_. Forty-eight hours later, the cells were trypsinized for direct mRNA expression analysis or seeded in 48-well plates (8 x10^4^ cells/well) for a cell traversal assay. After 24 hours, they were incubated with 10^5^ P*. yoelii* or *P. berghei* sporozoites for 2 hours or with 10^5^ P*. falciparum* sporozoites for 3 hours in the presence of rhodamine-dextran lysine fixable (10000 MW, Molecular Probes). After washing, the cells were trypsinized, fixed with 1% glutaraldehyde and analysed *via* FACS using an LSR Fortessa flow cytometer (BD Biosciences); 10^4^ cells were analysed (van Schaijk et al., 2008).

### Infection Inhibition Assay With the AQP9 Inhibitor Phloretin

To assess the function of AQP9 in *Plasmodium* parasite infection, we tested the effect of phloretin (Sigma, Saint Quentin Fallavier, France), an inhibitor that restricts the solute permeability of AQP9 (Tsukaguchi et al., 1998; Yang and Verkman, 2002; Liu et al., 2007), on infection of human primary hepatocytes by *P. falciparum*. Phloretin was co-incubated with the sporozoites in the first 2 hours of the invasion step. Thereafter, the cells were incubated with fresh complete medium for three additional days until EEF quantification. Phloretin concentrations up to 500 μM were tested because this concentration has previously been shown to inhibit 60% of urea and glycerol transport (Tsukaguchi et al., 1998) and to not affect rat hepatocyte viability (Mosmann, 1983; Huebert et al., 2002). Using an MTT toxicity assay (Mosmann, 1983), we assessed that phloretin was not toxic to primary human hepatocytes under the assay conditions **(** **Supplementary Figure 1** **).** We also assessed that phloretin was not toxic on *Plasmodium* sporozoites preincubated with different concentrations of phloretin **(** **Supplementary Figure 2** **)**.

### Data Analysis

GraphPad Prism 7 statistical Software (GraphPad. Software, San Diego, CA, USA) was used for data analysis and graphing. All values were expressed as means and standard deviations (SD). A *p-*value of 0.05 or less was considered as statistically significant.

## Results

### Comparative Transcriptomics of *Plasmodium falciparum* Permissive and Non-Permissive Hepatocytes Identified AQP9 as a Candidate Host Protein Involved in Sporozoite Invasion

**Figure 1:**
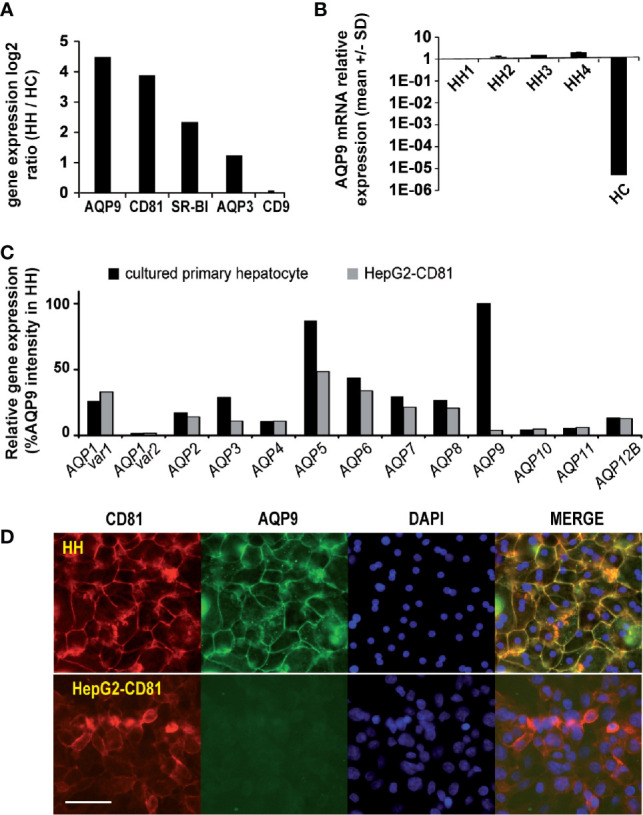
AQP9 and CD81 are expressed at higher levels in *P. falciparum* permissive human hepatocytes than in refractory hepatoma cells. **(A)** Transcriptomes of human hepatocytes (HHs) and human hepatoma HepG2-CD81 cells (HCs) were analysed using a 22K pan-genomic human oligo microarray (Agilent Technologies). AQP9, CD81, AQP3 and CD9 gene expression levels are presented as average log2 gene ratios in HH/HC. **(B)** RT-qPCR analysis of AQP9 mRNA expression in 4 sets of HHs and in HCs. Variations in transcript levels were calculated after normalisation to expression of the TBP gene and are presented relative to the AQP9 mRNA level in HH1. **(C)** Relative gene expression of aquaporins 1 to 12B in HHs from 3 donors and HCs is presented as the mean % of the AQP9 gene expression level in HHs, determined with the 22K pan-genomic human oligo microarray. **(D)** Immunofluorescence analysis of CD81 and AQP9 expression on HHs (upper panel) and HCs (lower panel). Cells were fixed, permeabilized, and incubated with a mixture containing a mouse monoclonal antibody against human CD81 and human AQP9 antibody and reacted with the appropriate secondary antibodies conjugated to Alexa 568 and Alexa 488, respectively. The white scale bar represents 100 µm.

To identify novel host factors and further understand human hepatocyte susceptibility to *P. falciparum* sporozoite invasion, we performed differential transcriptomic analysis of naturally *P. falciparum*-permissive human hepatocytes (HHs) and refractory human hepatoma HepG2-CD81 cells (HCs) using microarray technology. Only genes downregulated in non-permissive hepatoma cells were taken into account. Our results showed that a total of 276 genes were downregulated in non-permissive hepatoma cells compared to the permissive hepatocytes, with HH/HC ratios higher than 3. A list of the top 30 downregulated genes with the highest ratios is presented in **(** **Supplementary Table 2** **)**. As expected, *CD81* (with an HH/HC ratio of 14.7) was found to be among the most downregulated genes in hepatoma cells (**Figure 1A** and **Supplementary Figure 3A**). It should be noted that the *CD81* cDNA probe used on the DNA chips was designed to detect only endogenous *CD81* mRNA but not ectopic *CD81*. *SR-BI* (HH/HC=6) was also found amid the downregulated genes in hepatoma cells **(** **Figure 1A** and **Supplementary Figure 3A** **)**. In contrast, the expression ratio of the irrelevant *CD9* gene (HH/HC=1.06), which is known as not involved in hepatocyte permissiveness to *Plasmodium* infection (Yalaoui et al., 2008b), was not changed **(** **Figure 1A** and **Supplementary Figure 3A** **)**.

From evaluation of each candidate gene, *AQP9* which belongs to the aquaglyceroporin subfamily, a transmembrane channel protein that conducts water and glycerol (Liu et al., 2007), was selected for functional investigations, because it was among the most downregulated genes in the non-permissive HepG2 cells **(** **Figure 1A** and **Supplementary Figure 3A** **)**, and evidence from a previous study showed it to be involved in mouse susceptibility to *P. berghei* blood stage infection (Liu et al., 2007). Interestingly, another aquaglyceroporin, *AQP3*, which was reported to be involved in *P. berghei* development within human hepatoma cells and in *P. vivax* development within human primary hepatocytes but is not essential for sporozoite invasion (Posfai et al., 2018; Posfai et al., 2020), was not found to be significantly differentially expressed between the two cell types (HH/HC ratio of 2.3) **(** **Figure 1A** **)**. The relatively high *AQP9* gene expression in primary hepatocytes was further confirmed in hepatocytes isolated from 4 additional donors using qPCR (**Figure 1B**; **Supplementary Table 1**). The expression of other aquaporin genes was not or was only moderately downregulated **(** **Figure 1C** and **Supplementary Figure 3B** **)**. Moreover, immunofluorescence experiments showed that the drastic downregulation of the *AQP9* transcript in HepG2-CD81 cells was accompanied by a near total lack of protein expression **(** **Figure 1D** **)**. Overall, as a membrane-bound protein highly expressed in infection-permissive cells and almost silent in non-permissive cells **(** **Figure 1D** and **Supplementary Figure 4** **)**, hepatocyte AQP9 is a candidate protein that could be involved in sporozoite entry into cells.

### Aquaporin-9 Is Required for Efficient *P. falciparum* Sporozoite Entry Into Human Hepatocytes

To investigate the potential role of AQP9 in human hepatocyte infection by *P. falciparum* sporozoites, we performed *AQP9* gene silencing using five smalls interfering RNAs (siRNAs). These siRNAs were first tested for their ability to ablate *AQP9* gene expression in hepatocytes (**Supplementary Figure 5A** **)**. sihCD92 and sihCD81 were used as negative and positive controls, respectively. Gene silencing procedure as well as sihCD92 which are routinely used in our laboratory have no impact on *Plasmodium* infection. Next, using western blotting and quantitative immunofluorescence, we confirmed that siAQP9 does not affect CD81 protein levels (**Supplementary Figures 5A, B** **)**. Microscopic observation of the cultures transfected with either sihAQP9, sihCD81 or sihCD92 showed no obvious morphological alteration of the cells nor of the cell layer integrity (**Supplementary Figure 6** **)**. Finally, we checked the efficiency of the five siRNAs in blocking sporozoite infection of hepatocytes (**Supplementary Figure 5C** **)**. Silencing with sihAQP9^1,3,5^ resulted in an ~70% reduction in hepatocyte permissiveness to *P. falciparum* compared to the control sihCD92-treated hepatocytes. This reduction was similar to that obtained with sihCD81. sihAQP9^4^ caused an ~35% reduction in hepatocyte permissiveness, while sihAQP9^2^ had no effect. The reduction in hepatocyte infectivity observed with sihAQP9s was proportional to the inhibition of *AQP9* expression. Together, these results show that AQP9 and CD81 are individually required for *Plasmodium* infection but that neither is able to compensate for silencing of the other. We choose siAQP9^1^ among the 3 siRNA (siAQP9 ^1,3^ and ^5^) showing a potent and comparable silencing of AQP9 protein to be used for the rest of our experiments.

**Figure 2:**
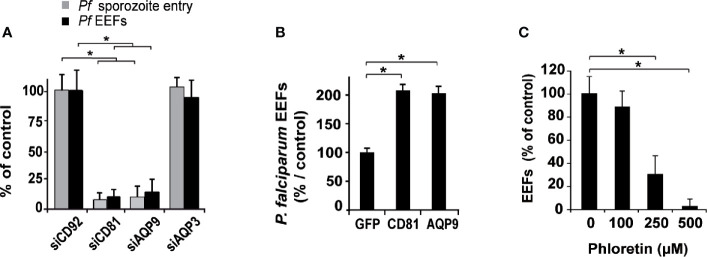
Human hepatocyte permissiveness to *P. falciparum* sporozoite infection depends on AQP9 expression. **(A)** Hepatocytes were transfected with sihRNAs and tested for their permissiveness to *P. falciparum* sporozoite infection: sihCD92-, sihCD81-, sihAQP9^1^- and sihAQP3-transfected human hepatocytes were infected three days after transfection with *P. falciparum* sporozoites and cultured for 3 hours to evaluate sporozoite entry *via* double immunostaining for *Pf*CSP protein (grey bars) or for four days to evaluate cell infection after immunolabelling of EEFs using HSP70 antibody (black bars). Sporozoite and EEF numbers are expressed as a percentage of those in sihCD92-transfected control hepatocytes. *p = 0.0006. **(B)** Hepatocytes overexpressing CD81 or AQP9 were tested for their permissiveness to *P. falciparum* infection: hepatocytes transduced with a lentiviral vector expressing GFP protein, as a control, hCD81, or hAQP9 were infected three days later with sporozoites and cultured for four days until EEF quantification. EEF numbers are expressed as a percentage of those in GFP-transduced control hepatocytes. The mean schizont numbers per well in controls of three independent experiments were 141, 203, and 314. *p = 0.05. **(C)** Hepatocytes were treated for the first two hours following sporozoite inoculation with three doses of phloretin. Hepatocytes were then washed and incubated with fresh complete medium for three additional days until EEF quantification. EEF rates are relative to those in untreated hepatocytes. *p = 0.0009. All analyses were carried out in triplicate wells, and the results are expressed as the mean ± standard deviation. *p* values were determined by Mann-Whitney U test.

To further define the role of AQP9, we investigated its specific contribution to sporozoite entry into hepatocytes. Hepatocytes were inoculated with *P. falciparum* sporozoites three days after transfection, and their entry was evaluated by double immunostaining three hours post-sporozoite infection (Rénia et al., 1988; Silvie et al., 2002). The entry of parasites into sihAQP9- and sihCD81-treated hepatocytes was decreased by 90% and 92%, respectively, compared to sihCD92-treated cells **(** **Figure 2A** and **Supplementary Figure 7A** **)**, and this inhibitory effect was similar to that observed for infection efficiency. In contrast, *AQP3* gene silencing did not affect *Plasmodium* sporozoite infection or entry **(** **Figure 2A** and **Supplementary Figure 7A** **)**, corroborating results obtained elsewhere (Posfai et al., 2018).

We then examined whether AQP9 overexpression might improve cell permissiveness to *P. falciparum* sporozoite infection, as overexpression of CD81 in hepatocytic cell lines did for rodent *Plasmodium* species (Silvie et al., 2006b). To this end, we transduced human hepatocytes that naturally express endogenous *CD81* and *AQP9* with a lentiviral vector allowing expression of *CD81* or *AQP9*, and then cultured the hepatocytes for three days prior to *Plasmodium* sporozoite infection. Cells transduced with a vector encoding the irrelevant GFP protein were used as a control (*GFP* expression in liver cells was previously reported to not alter *Plasmodium* infection efficiency (Yalaoui et al., 2008b)). Overexpression of *AQP9* (as assessed by western blotting, **Supplementary Figure 8**) induced a two-fold increase in hepatocyte permissiveness to *P. falciparum* infection compared with GFP-transduced hepatocytes, comparable to that of CD81 **(** **Figure 2B** **)**. Pharmacologic inhibition of AQP9 by phloretin during the first two hours of hepatocyte co-incubation with sporozoites resulted in a significant reduction in hepatocyte infection in a dose-dependent manner (**Figure 2C**). We also found that cell traversal of *P. falciparum* sporozoites was not affected by either *CD81* or *AQP9* silencing (**Supplementary Figure 9A**).

Since *AQP9* RNA silencing induced a major, but incomplete, inhibition of *P. falciparum* infection, with 10 to 30% residual development of liver schizonts, we could assess whether AQP9 might play a role in the maturation of the parasite. SihAQP9^1^-treated human hepatocytes infected by *P. falciparum* sporozoites were labelled four days post-inoculation with an anti-PfHSP70 antibody in order to measure the size of the schizonts. Fluorescence microscopy analysis revealed that schizont diameters were similar in sihCD92, sihCD81 and sihAQP9^1^-treated hepatocytes **(** **Supplementary Figure 10A**).

Taken together, these data clearly provide evidence that *AQP9* expression is important for *P. falciparum* sporozoite entry into human hepatocytes but have no influence on parasite development at the liver stage. Interestingly, the magnitude of the effect of *AQP9* silencing in human hepatocytes on infection by *P. falciparum*, either on parasite entry or on the number of schizonts, is comparable to that of *CD81* silencing. Similarly, both *AQP9* overexpression and *CD81* overexpression significantly enhance *P. falciparum* infection of human hepatocytes.

### The Rodent Parasites *P. yoelii* and *P. berghei* Differentially Depend on AQP9 for Infection of Liver Cells

**Figure 3:**
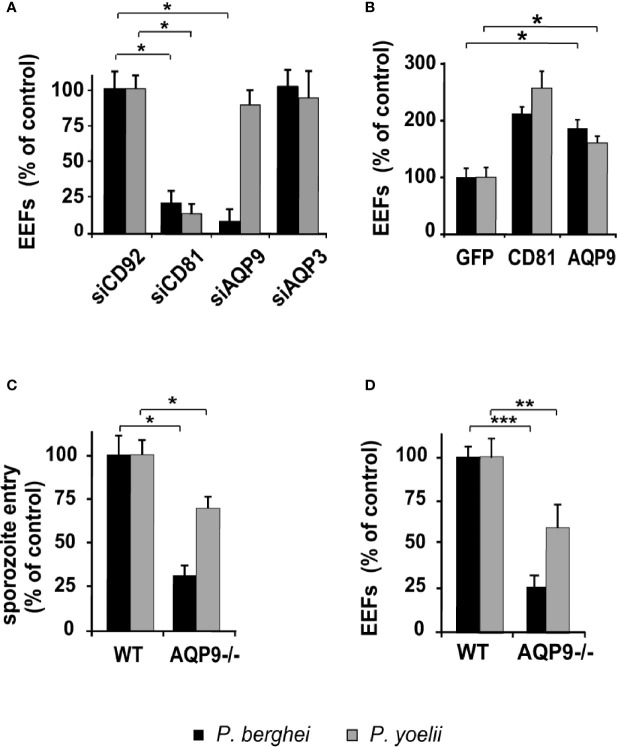
Hepatocyte permissiveness to rodent parasites differentially depends on AQP9 according to parasite/host cell combinations. **(A)** sihRNA-transfected human hepatocytes (HHs) were infected with sporozoites three days post-transfection and cultured for two days until EEF quantification. EEF values are expressed as a percentage of those in control sihCD92-transfected hepatocytes. *p = 0.0001. **(B)** Three days post lentiviral transduction, HHs overexpressing GFP protein, hCD81, or hAQP9 were cultured for two days until EEF quantification. EEF numbers are expressed as a percentage of those in control GFP-transduced hepatocytes. *p = 0.0079. **(C, D)** Primary hepatocytes isolated from wild-type (WT) and AQP9 knockout (AQP9-/-) mice were infected with *P. berghei* or *P. yoelii*. Sporozoite cell entry was evaluated 3 h post-infection using double immunostaining **(C)**, whereas EEF quantification was performed after 48 h of culture **(D)**. Penetration rates and EEF numbers are expressed as a percentage of those in control WT hepatocytes. *p = 0.05; **p = 0.0018; ***p = 0.0004. The results are expressed as the mean ± standard deviation. *p* values were determined by Mann-Whitney U test.

We further extended our experiments to study liver infection by the rodent malaria parasites *P. yoelii* and *P. berghei*. Given that the requirement for host proteins that confer permissiveness to *Plasmodium* infection may differ according to the species of the target cell (Silvie et al., 2007) and that *P. yoelii* and *P. berghei* sporozoites are capable of infecting human hepatocytes *in vitro* (Silvie et al., 2006b), the role of AQP9 was investigated in infection of human hepatocytes by these rodent *Plasmodium* species. The permissiveness of sihCD81- and sihAQP9^1^-treated human hepatocytes to *P. berghei* infection was reduced by~79% and ~92%, respectively. As expected, silencing of *CD81* reduced *P. yoelii* infection by ~87%, but surprisingly, silencing of *AQP9* did not affect *P. yoelii* infection **(** **Figure 3A** and **Supplementary Figure 7B** **)**. In fact, the dramatic effect of *AQP9* silencing on infection by *P. berghei* was also somewhat unexpected, because it is well known that human HepG2 hepatoma cells can be readily infected by this parasite, despite undetectable *AQP9* expression. This paradox was further investigated in HepaRG cells that express *AQP9*. *P. berghei* infection of these cells was markedly decreased by *AQP9* RNA silencing **(** **Supplementary Figures 11A, B** **)**, which suggests that this parasite uses AQP9-dependent pathways to invade human liver cells naturally expressing *AQP9* but is able to use alternative pathways in hepatoma cells that do not express AQP9 (see **Table 1**). *CD81* or *AQP9* overexpression in human hepatocytes transduced with CD81- or AQP9-expressing lentiviral vectors led to an increase in the number of liver schizonts in each rodent species (**Figure 3B**). Finally, the availability of AQP9 knockout mice prompted us to test permissiveness of primary hepatocytes isolated from wild-type or AQP9 knockout mice to rodent *Plasmodium* sporozoites. The total lack of AQP9 resulted in a reduction in hepatocyte permissiveness to *P. berghei* and *P. yoelii* infection by ~77% and ~34%, respectively **(** **Figure 3C** **)**. Sporozoite entry was reduced by ~69% and ~30% for *P. berghei* and *P. yoelii* sporozoites, respectively **(** **Figure 3D** **)**. Similar to *P. falciparum*, *AQP9* silencing had no effect on cell traversal (**Supplementary Figure 9B** **)**. *P. berghei* schizont diameters measured two days post-infection were similar in AQP9-knockout and wild type hepatocytes (**Supplementary Figure 10B**) thus confirming that AQP9 is not involved in parasite development at the liver stage.

**Table 1:** Summary of the role of AQP9 in susceptibility to *Plasmodium* infection according to the different cell types and plasmodial species tested on the basis of RNA silencing experiments.

	Human				Mouse
	Human hepatocytes (CD81+/AQP9+)	HepG2 (CD81^-/^AQP9^-^)	HepG2/CD81(CD81^+/^AQP9^-^)	HepaRG(CD81+/AQP9+)	Mouse hepatocytes (CD81^+/^AQP9^+^)
***P. falciparum***	AQP9-dependent****	no infection	no infection	infection (role of AQP9 not tested)	no infection
***P. yoelii***	AQP9-independent	no infection	AQP9-independent	infection (role of AQP9 not tested)	AQP9-independent
***P. berghei***	AQP9-dependent****	AQP9-independent	AQP9-independent	AQP9-dependent****	AQP9-dependent****

Altogether, these results indicate that *P. berghei* and *P. yoelii* display differential dependence on AQP9 for entry into hepatocytes. Indeed, *P. yoelii* invasion is found to not be or be poorly AQP9-dependent, whereas for *P. berghei*, invasion depends mainly on AQP9, at least in human liver cells that express this protein.

## Discussion

The first multiplicative stage of malaria parasites within their human host occurs exclusively in the hepatocyte *in vivo*, and this development can also be obtained *in vitro*. It is interesting that CD81-expressing human hepatoma HepG2 cells are permissive to productive infection by sporozoites of the rodent parasites *P. berghei* and *P. yoelii*, but not to productive infection by *P. falciparum* (Silvie et al., 2006a). This differential susceptibility was exploited to search for proteins responsible for the exclusive permissiveness of human hepatocytes to *P. falciparum* sporozoites. Comparison of the transcriptome of human hepatocytes with that of human hepatoma HepG2-CD81 cells, which do not support *P. falciparum* sporozoite infection, led to identification of genes whose expression is downregulated in hepatoma cells. We focused our functional studies on AQP9, a membrane protein already suspected to be involved in the virulence of the rodent parasite *P. berghei* (Liu et al., 2007) and whose gene expression was one of those most downregulated in hepatoma cells. Our results demonstrate that AQP9 is required for efficient *P. falciparum* sporozoite entry into human hepatocytes. Furthermore, we found that the rodent parasites *P. yoelii* and *P. berghei* differentially depend on AQP9 and that this difference varies depending on the host cell type.

AQP9 is a member of the AQPs family, which consists of 13 distinct small hydrophobic membrane proteins with predominant roles in trans-cellular water transport in response to osmotic gradients (Verkman, 2005). Among the aquaporins, AQPs 3, 7, 9 and 10, referred to as aquaglyceroporins, also transport glycerol and other small uncharged solutes (Verkman, 2005). Human *AQP9* is widely expressed in the liver but also in other tissues, including the lung, spleen and leukocytes, whereas rodent AQP9 has mainly been found at the basolateral hepatocyte plasma membrane facing the sinusoids (Huebert et al., 2002; Mazzone et al., 2006) and in a less abundant manner in other tissues, such as the testis, epididymis, spleen, brain and lung (Ishibashi et al., 1998; Tsukaguchi et al., 1998; Elkjaer et al., 2000). By regulating water entry or entry of other small uncharged solutes and interacting with cytoskeleton elements and signalling cascades, the AQPs can influence cell morphology, volume, motility and migration (Saadoun et al., 2005; Holm et al., 2016). Therefore, they are considered potential therapeutic targets (Saadoun et al., 2005).

The results of our gene silencing experiments demonstrate that AQP9 expression is required for *P. falciparum* sporozoite infection of human hepatocytes, and this requirement appears to be of the same order of magnitude as it is for CD81, which thus far is the only hepatocyte surface molecule known to be strictly required for *P. falciparum* sporozoite infectivity (Silvie et al., 2003). Moreover, overexpression of AQP9, as well as of CD81, in human hepatocytes led to increased *P. falciparum* infection. From our previous studies on CD81 (Silvie et al., 2003) and the current results presented in this paper, one can conclude that *P. falciparum* require both CD81 and AQP9 to invade hepatocytes as the invasion is drastically hampered in the absence of one or other. There are two arguments that support this conclusion. First, diminishing expression levels of one does not impact on the other one. Second, the reduction in hepatocyte infectivity observed with the inhibition of the expression of either protein was proportional to its inhibition level, as none is able to compensate for silencing of the other, suggesting that these two proteins are operating independently and/or sequentially. While not tested here, it would be interesting to test whether simultaneous silencing of both AQP9 and CD81 proteins could result in a complete invasion-inhibition.

Additional experiments using the rodent parasites *P. yoelii* and *P. berghei* showed that the requirement for AQP9 varies with the species of the parasite and of the host cell. Thus, invasion of *P. berghei* sporozoites was more dependent on the presence of AQP9 than invasion of *P. yoelii* sporozoite in murine hepatocytes, and this effect was more pronounced when human hepatocytes were used. These findings differ from those obtained for CD81 dependence (Silvie et al., 2007). Indeed, when human hepatocytes are used, *P. yoelii* invasion is strictly dependent on CD81 but only poorly dependent on AQP9, whereas for *P. berghei*, invasion is mainly dependent on AQP9, at least in human liver cells that express this protein, as it is on CD81. It should be noted that *P. berghei* is strictly CD81-dependent for invasion of the mouse cell line Hepa 1.6 but CD81-independent when human hepatoma cell lines are used (Silvie et al., 2007).

Taken together, our results showed that AQP9 and CD81 are individually required for *Plasmodium* sporozoite infection, but that neither is able to compensate for the absence of the other. Moreover, the species specificity for *P. falciparum* and *P. berghei*, with a lesser impact on *P. yoelii*, suggest a distinct role for AQP9 to the functions of previously described CD81 or SR-BI (Silvie et al., 2003; Yalaoui et al., 2008a; Risco-Castillo et al., 2014; Manzoni et al., 2017), probably acting downstream to the events requiring both these host proteins. Furthermore, our findings strengthen previous observations that sporozoite entry into liver cells is a complex phenomenon and suggest that extrapolation of results obtained with rodent models to human parasites should be carried out with caution. This complexity is consistent with the general view that entry of intracellular pathogens relies on plastic and dynamic interactions between various molecules at the cell surfaces of the pathogen and host, as illustrated for HIV, HCV, and *P. falciparum* merozoites (Cosset and Lavillette, 2011; Bartholdson et al., 2013) and for the role of CD81, SR-BI and CD9P-1 in hepatocyte invasion by *Plasmodium* sporozoites (Yalaoui et al., 2008a; Charrin et al., 2009).

Our experiments with human and rodent parasites showed that AQP9 plays a crucial role in sporozoite invasion but not in the cell traversal that precedes it. Similar results were reported for CD81, which was also found to not be required for migration of *P. falciparum* and *P. yoelii* sporozoites through cells (Silvie et al., 2003). Internalization of the *Plasmodium* sporozoite and the associated PVM formation through invagination of the host cell plasma membrane involves a series of finely orchestrated events, including gliding motility, moving junction formation, and penetration (Dundas et al., 2019). Several studies have shown that this invasion process, as is the case in other Apicomplexa parasites, requires not only the parasite’s actin-myosin motor but also membrane reorganization and *de novo* polymerization of host actin at the entry site for anchoring the junction on which the parasite pulls to penetrate the host cell (Gonzalez et al., 2009; Gomes-Santos et al., 2012; Gaji et al., 2013). Interestingly, previous studies have shown that AQP9 plays a major role in processes involving dynamic membrane reorganization and actin polymerization (Loitto et al., 2002; Loitto et al., 2007). More importantly, studies of AQP9 inhibition have shown that it promotes cell membrane protrusions through its water transport function. It has been proposed that AQP9-induced water influx results in a pressure-driven forward push of the membrane, creating a gap that allows diffusion and polymerization of actin monomers (Loitto et al., 2009; Karlsson et al., 2013). In our study, the inhibitory effect observed with phloretin strongly suggests an important role for the transport of water, and possibly of other small molecules, in liver cell invasion by *Plasmodium* sporozoites. Although it is not clear how AQP9 functions to promote sporozoite invasion into hepatocytes, one could speculate that AQP9 might play an indirect role through actin polymerization, possibly through its water transport function. Interestingly, a similar mechanism has been reported for *Cryptosporidium parvum*, another intracellular apicomplexan parasite, in which the host aquaporin AQP1 has been shown to be crucial for entry of the parasite into cholangiocytes (Chen et al., 2005). More specifically, it was shown that AQP1 mediates the localized water influx that leads to the increase in cell volume and actin polymerization accompanying the membrane protrusion that allows *C. parvum* entry and PVM formation (Chen et al., 2005). Collectively, these data support a model in which AQP9, through its water permeation function and possibly permeation of other small molecules, induces the membrane reorganization and actin polymerization, likely at the entry site, required to permit invagination of the PVM and entry of the sporozoite. Additional studies will be necessary to confirm this hypothesis.

In conclusion, the present study demonstrates that the host protein AQP9 is involved in entry of *P. falciparum* (and of *P. berghei*) sporozoites into hepatocytes and suggests that this process may involve water transport and possibly other small molecules. Our findings also suggest that *P. falciparum* sporozoites are able to highjack hepatocyte membrane machinery to achieve internalization into the parasitophorous vacuole. Collectively, our data provide new insights into the complex cellular process required for *Plasmodium* infection of liver cells and may assist in the development of host factor-directed antimalarial therapies.

## Data Availability Statement

The original contributions presented in the study are included in the article/**Supplementary Material**. Further inquiries can be directed to the corresponding author.

## Ethics Statement

The protocol was approved by the Ethics Committee for Animal Experiments of University Pierre et Marie Curie, Paris 6, France (Permit Number: 75-1087).

## Author Contributions 

NA, J-FF, ST, SY, PF, and DM conceived, planned, and designed experiments. NA, J-FF, ST, SY, AL, VS, AR, AG, VR-C and MT conducted experiments. NA, J-FF, ST, SY, AL, VS, AG, AR, VR-C, AM, MT, G-JG, RS, J-CV, PR, J-LP, PF, and DM analyzed the data. J-FF, PF, G-JG, RS, J-CV, PR and DM provided essential materials. NA, J-FF, ST, SY, PF, J-LP and DM wrote the manuscript. All authors contributed to the article and approved the submitted version.

## Funding

SY was supported by a fellowship from the European Community. This work was supported in part by grants from the Agence Nationale pour la Recherche (ANR-6 BLAN-0378 and MaTuRe project) and the European Community (LSHP-CT-2005-012199/MALINV).

## Conflict of Interest

The authors declare that the research was conducted in the absence of any commercial or financial relationships that could be construed as a potential conflict of interest.
