# Supplementary material for: The Host Protein Aquaporin-9 is Required for Efficient Plasmodium falciparum Sporozoite Entry into Human Hepatocytes
Source: Front Cell Infect Microbiol. 2021 Jun 29;11:704662. doi: 10.3389/fcimb.2021.704662 (PMC8276244; doi:10.3389/fcimb.2021.704662)
Supplement: Supplementary file 1 [file DataSheet_1.docx]

**Supplementary Figures and Tables**

**Supplementary Table 1. Information about the donors of the liver fragments used for microarray and RT-qPCR analyses**

| **Gene expression analysis** | **Sample** | **Age of the patient** (years) | **Sex** | **Indication of partial hepatectomy** |
| --- | --- | --- | --- | --- |
| DNA chip normalization | Liver | 67 | F | Metastatic colorectal cancer |
| DNA chip hybridizations | HH-A | 59 | F | Hepatic adenoma |
|  | HH-B | 62 | F | Liver metastasis of undetermined origin |
|  | HH-C | 51 | F | Metastatic colorectal cancer |
| RT-qPCR AQP9 quantification | HH1 | 62 | M | Metastatic colorectal cancer |
|  | HH2 | 63 | F | Metastatic colorectal cancer |
|  | HH3 | 51 | F | Metastatic stomach cancer |
|  | HH4 | 64 | M | Metastatic colorectal cancer |
| F: female; M: male; HH: human hepatocyte | | | | |

**Supplementary Table 2.** **List of top downregulated genes in *P. falciparum-*nonpermissive human hepatoma HepG2-CD81 cells (HCs) compared to permissive human hepatocytes (HHs) identified by transcriptomic analysis**

| **Acc. ID** | **GeneName** | **Description** | **Mean HepG2/Ref** | **Mean HH/ref** | **Mean HH/HepG2** |
| --- | --- | --- | --- | --- | --- |
| NM_001482 | GATM | Glycine amidinotransferase (L-arginine:glycine amidinotransferase) | 0,02 | 0,96 | 53,46 |
| NM_000764 | CYP2A7 | Cytochrome P450, family 2, subfamily A, polypeptide 7 | 0,03 | 1,40 | 51,93 |
| NM_000893 | KNG1 | Kininogen 1 | 0,03 | 1,26 | 44,46 |
| NM_000762 | CYP2A6 | Cytochrome P450, family 2, subfamily A, polypeptide 6 | 0,03 | 1,13 | 36,08 |
| NM_004132 | HABP2 | Hyaluronan binding protein 2 | 0,06 | 1,87 | 29,35 |
| NM_004617 | TM4SF4 | Transmembrane 4 L six family member 4 | 0,05 | 1,43 | 27,49 |
| NM_000638 | VTN | Vitronectin (serum spreading factor, somatomedin B, complement S-protein) | 0,05 | 1,16 | 23,01 |
| NM_000583 | GC | Group-specific component (vitamin D binding protein) | 0,02 | 0,35 | 22,80 |
| NM_020980 | AQP9 | Aquaporin 9 | 0,02 | 0,47 | 22,31 |
| NM_018487 | HCA112 | Hepatocellular carcinoma-associated antigen 112 | 0,02 | 0,47 | 21,66 |
| NM_001076 | UGT2B15 | UDP glucuronosyltransferase 2 family, polypeptide B15 | 0,08 | 1,72 | 21,22 |
| NM_002506 | NGFB | Nerve growth factor, beta polypeptide | 0,03 | 0,63 | 19,78 |
| NM_006149 | LGALS4 | Lectin, galactoside-binding, soluble, 4 (galectin 4) | 0,31 | 5,97 | 19,44 |
| NM_001014446 | OCIAD2 | OCIA domain containing 2 | 0,23 | 4,21 | 18,32 |
| NM_000040 | APOC3 | Apolipoprotein C-III | 0,10 | 1,79 | 17,30 |
| NM_000613 | HPX | Hemopexin | 0,01 | 0,20 | 17,15 |
| NM_000505 | F12 | Coagulation factor XII (Hageman factor) | 0,08 | 1,36 | 16,80 |
| NM_000766 | CYP2A13 | Cytochrome P450, family 2, subfamily A, polypeptide 13 | 0,13 | 2,04 | 16,28 |
| NM_001701 | BAAT | Bile acid Coenzyme A: amino acid N-acyltransferase (glycine N-choloyltransferase) | 0,03 | 0,53 | 15,91 |
| NM_007283 | MGLL | Monoglyceride lipase | 0,14 | 2,22 | 15,81 |
| NM_153187 | SLC22A1 | Solute carrier family 22 (organic cation transporter), member 1 | 0,06 | 0,85 | 15,11 |
| NM_015714 | G0S2 | Putative lymphocyte G0/G1 switch gene | 0,05 | 0,83 | 15,01 |
| NM_004356 | CD81 | Tetraspanin CD81 | 0,09 | 1,36 | 14,71 |
| NM_000772 | CYP2C18 | Cytochrome P450, family 2, subfamily C, polypeptide 18 | 0,08 | 1,07 | 13,25 |
| NM_198336 | INSIG1 | Insulin induced gene 1 | 0,04 | 0,49 | 13,21 |
| NM_002444 | MSN | Moesin | 0,24 | 3,10 | 12,90 |
| NM_001639 | APCS | Amyloid P component, serum | 0,01 | 0,17 | 12,81 |
| NM_001909 | CTSD | Cathepsin D (lysosomal aspartyl protease) | 0,06 | 0,80 | 12,66 |
| NM_000030 | AGXT | Alanine-glyoxylate aminotransferase | 0,02 | 0,28 | 12,66 |
| NM_001072 | UGT1A6 | UDP glucuronosyltransferase 1 family, polypeptide A6 | 0,05 | 0,57 | 12,58 |

**
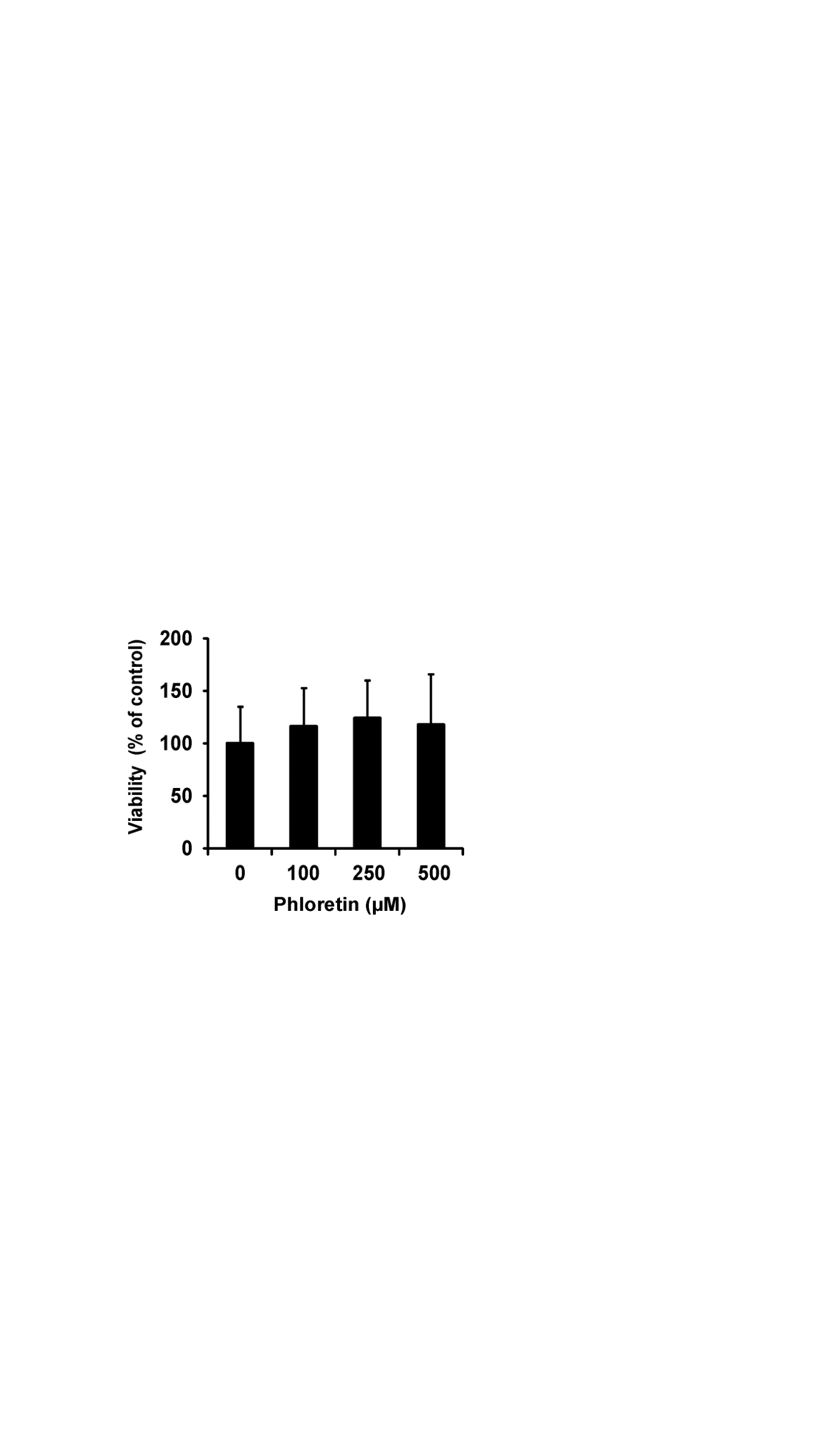
**

**Supplementary Figure 1. Toxicity of Phloretin towards human hepatocytes.** Hepatocytes were treated for two hours with 100 μM to 500 μM phloretin. Phloretin was then washed out, and the cells were incubated with fresh complete medium for two additional days. Viability was assessed with MTT assays as previously described in Mahnoudi N 2006. Absorbance was read at 570 nM on a Flex Station, and data are shown as a percentage of the absorbance of the untreated control, the average of 3 technical replicates is presented. Error bars represent standard deviation; one representative experiment of two independent experiments is shown.


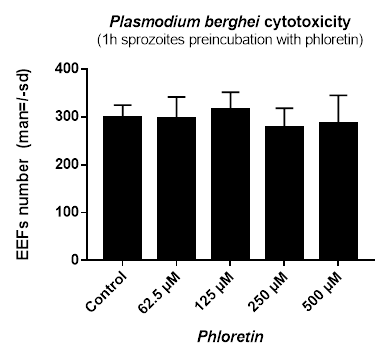


**Supplementary Figure 2. Toxicity of phloretin towards *Plasmodium* sporozoites.** Freshly dissected *P. berghei* sporozoites were preincubated with varying concentrations of phloretin for 1 hour at room temperature. The drug was then removed by centrifugation and sporozoites resuspended in culture medium before being allowed to infect hepatocytes. The infected hepatocytes were incubated with fresh complete medium for two additional days before EEFs quantification to evaluate toxicity. Analyses were carried out in triplicate wells, and the results are expressed as the mean ± standard deviation.


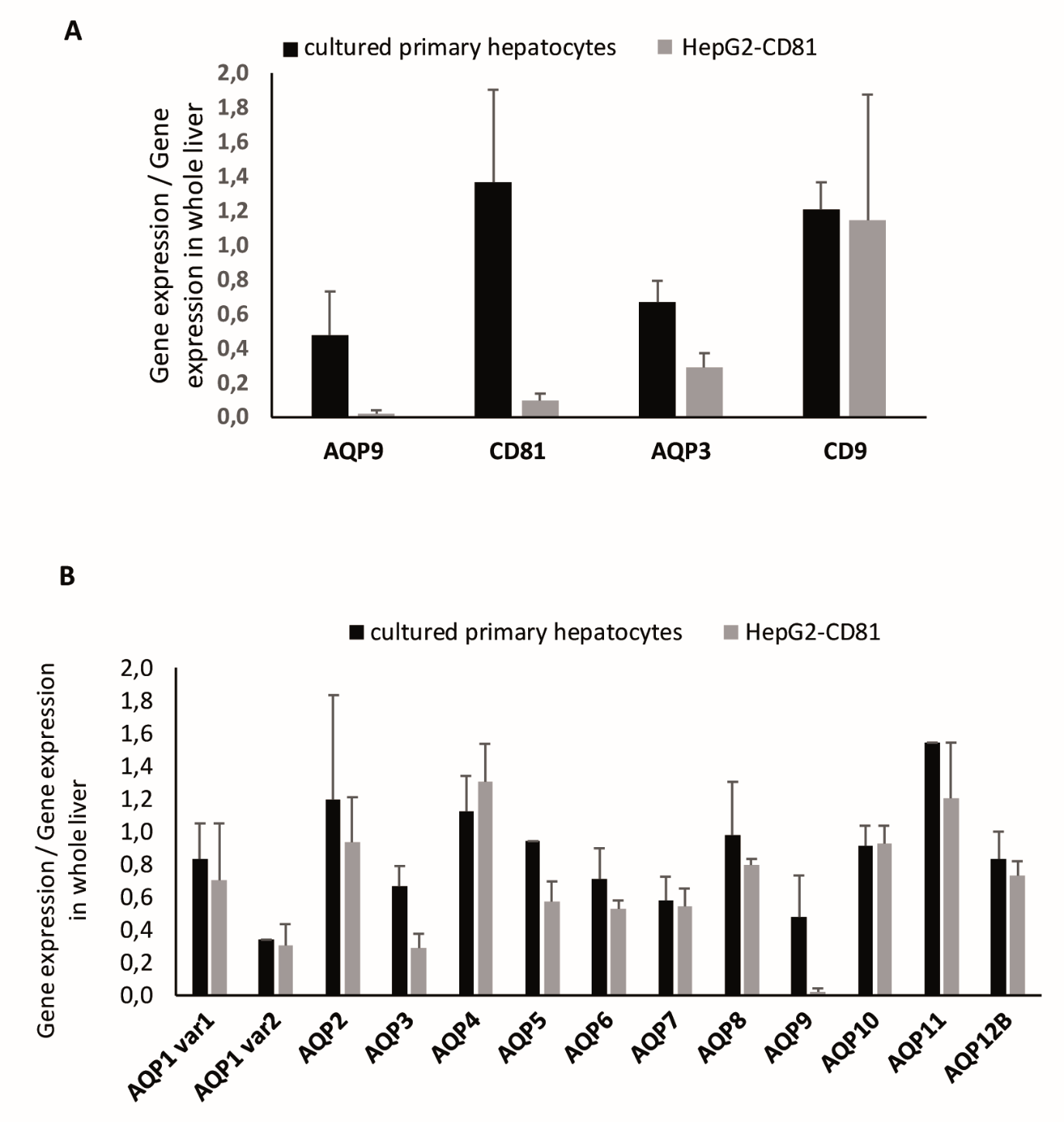


**Supplementary Figure 3. AQP9 and CD81 are expressed at higher levels in *P. falciparum* permissive human hepatocytes than in refractory hepatoma cells. (A)** Transcriptomes of human hepatocytes (HHs) and human hepatoma HepG2-CD81cells were analyzed using a 22K pan-genomic human oligo microarray (Agilent Technologies). AQP9, CD81, AQP3 and CD9 gene expression levels are presented as the mean ratio of three independent hybridizations using different hepatocyte donors and HepG2-CD81 samples, compared to the expression level of the reference RNA (whole human liver RNA) used for all hybridizations. **(B)** Relative gene expression of aquaporins 1 to 12B in HHs from 3 donors and HCs is presented as the mean ratio of three independent hybridizations using different hepatocyte donors and HepG2-CD81samples, compared to the expression level of the reference RNA (whole human liver RNA) used for all hybridizations, determined with the 22K pan-genomic human oligo microarray.


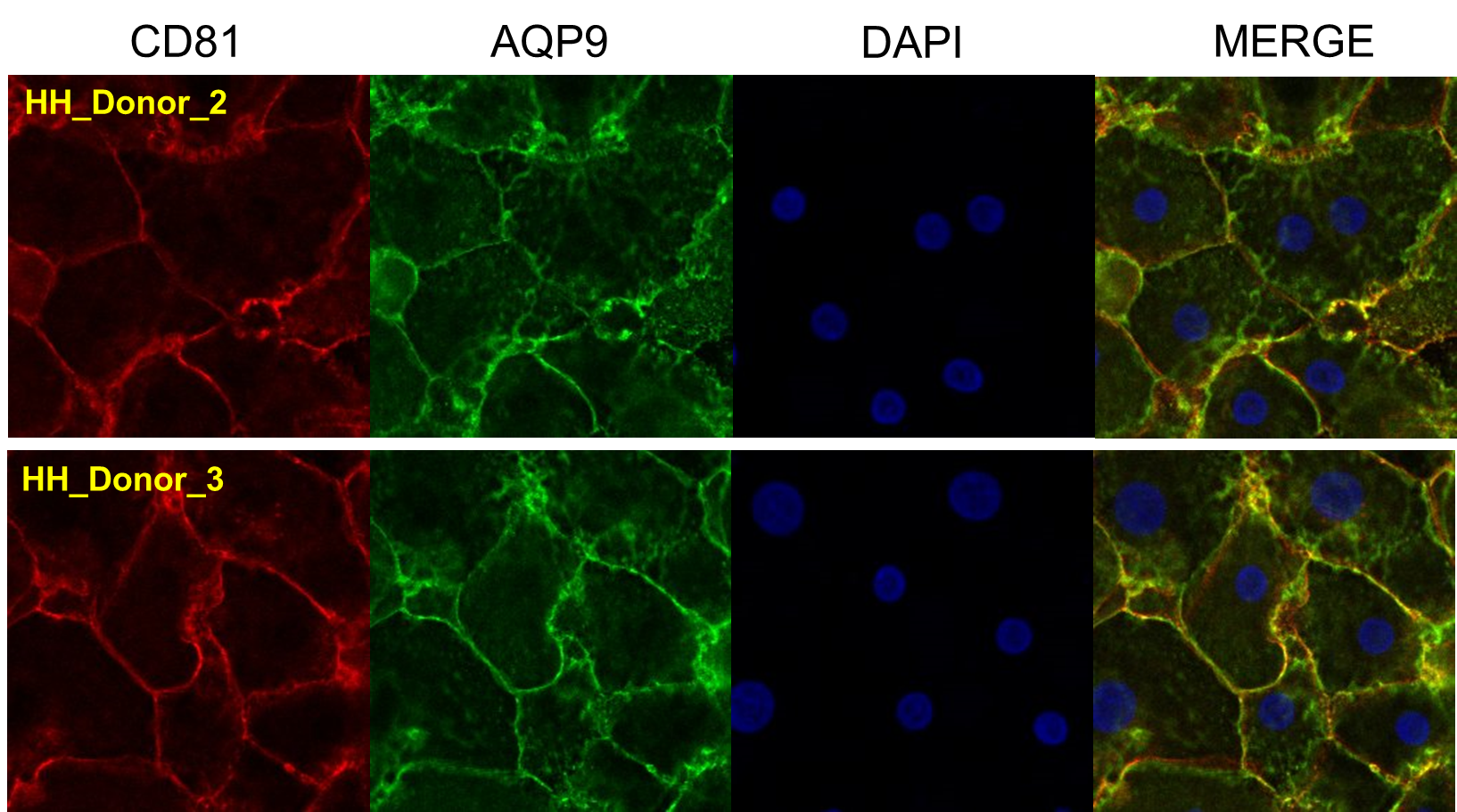


**Supplementary Figure 4. Immunofluorescence analysis of CD81 and AQP9 expression on primary human hepatocytes from different donors.**


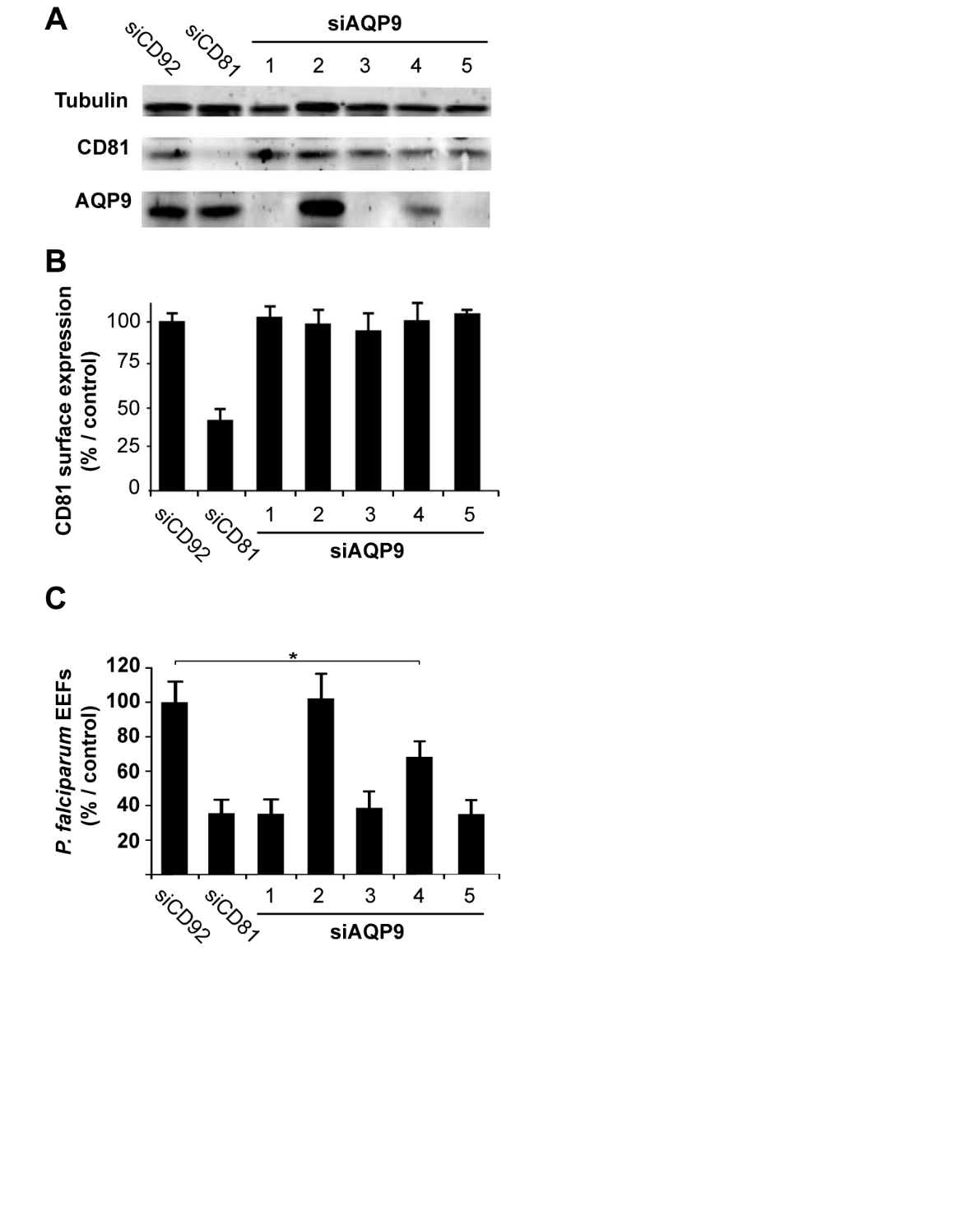


**Supplementary Figure 5. Selection and confirmation of siRNAs targeting AQP9.** Five sihAQP9s (sihAQP9_1-5_) were tested for their ability to inhibit AQP9 expression and block sporozoite entry. **(A)** sihAQP9_1-5_-, sihCD81- and sihCD92-transfected hepatocytes were lysed 72 hours post-transfection, and cell lysates were loaded onto a 12% non-reducing SDS-PAGE gel. CD81 and AQP9 expression levels in whole cells were determined by immunoblotting. **(B)** siRNA-transfected hepatocytes were analysed for CD81 surface expression via quantitative immunofluorescence three days post-transfection. Values are expressed as a percentage of that in sihCD92-transfected control hepatocytes. **(C)** siRNA-treated human hepatocytes were tested for their permissiveness to infection by *P. falciparum* sporozoites. Cells were infected with *P. falciparum* sporozoites 72 hours post-transfection and cultured for four additional days before exo-erythrocytic form (EEF) quantification. EEF numbers are expressed as a percentage of those in control sihCD92-transfected hepatocytes. Analyses were carried out in triplicate wells, and the results are expressed as the mean ± standard deviation. The mean schizont numbers per well in controls of two independent experiments were 145.5 and 216. *: *p*=0.0126, determined by Mann-Whitney U test. All data are representative of two independent experiments using freshly isolated hepatocytes from two different donors.

**
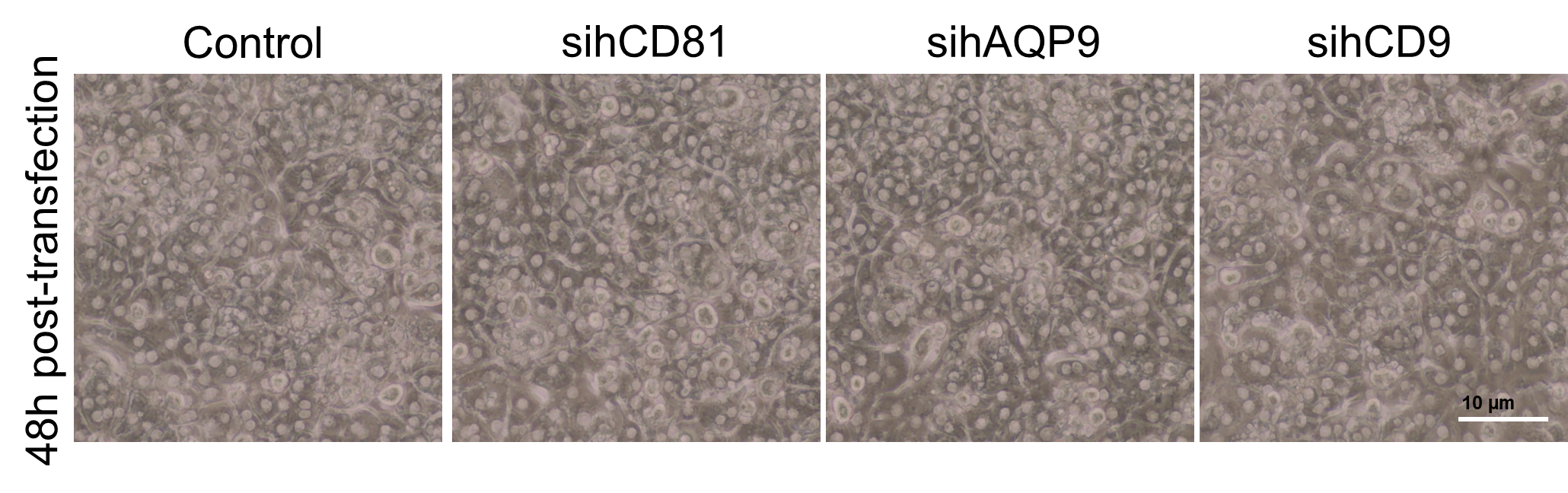
**

**Supplementary Figure 6. Light microscopy images showing normal morphologies of hepatocyte cultures following transfection with different siRNAs and non-transfected control cells.**


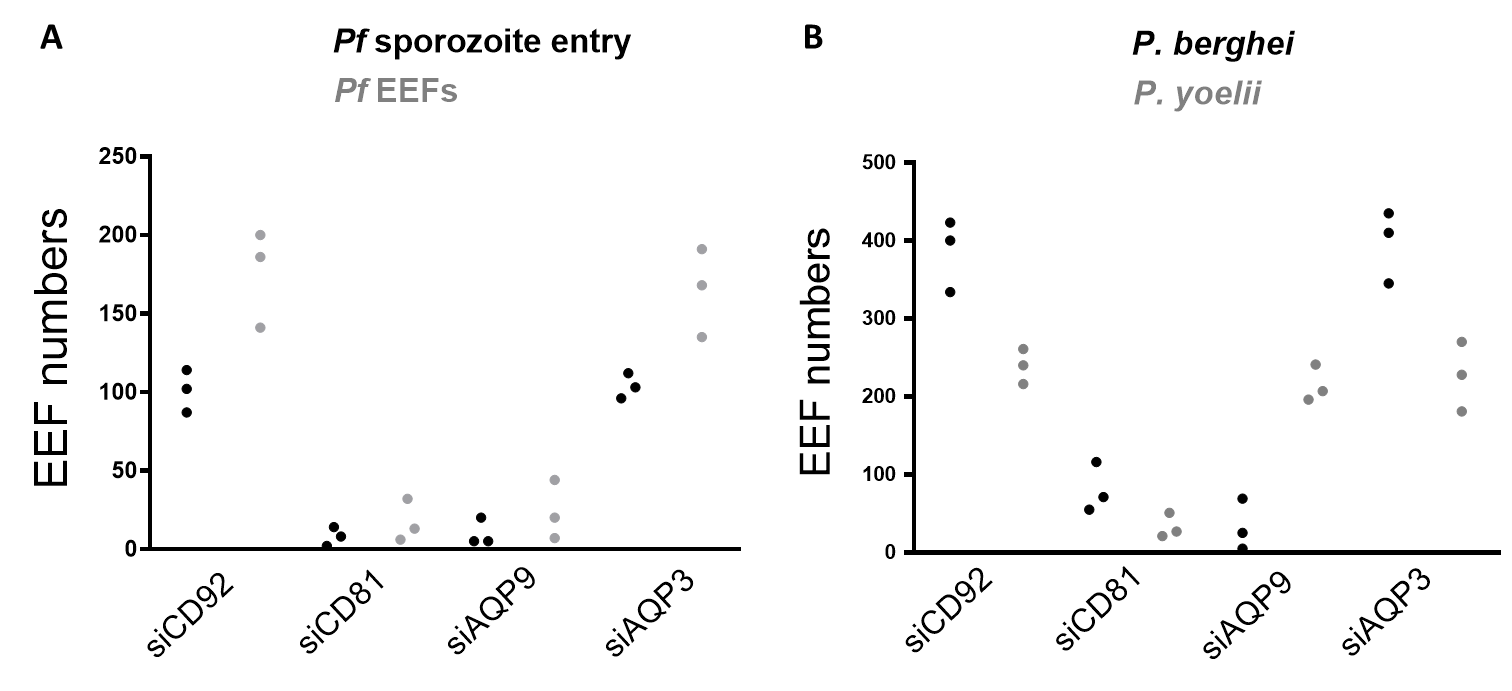


**Supplementary Figure 7. Raw Data for figures 2A and 3A.** (A) Each point corresponds to either the number of intracellular sporozoites counted 3 hours post infection in sihRNAs transfected cultures to evaluate sporozoite entry (grey dots) or corresponds to exoerythrocytic forms (black dots) counted in each well 4 days post-infection with sporozoite. These data were used to draw figure 2A by averaging each condition and normalizing to the mean values obtained with siCD92 control siRNA. (B) Each point corresponds to either the number of exoerythrocytic forms of *P. berghei* (black dots) or *P. yoelii* (grey dots) counted in each well 2 days post-infection with sporozoite in sihRNAs transfected cultures. These data were used to draw figure 3A by averaging each condition and normalizing to the mean values obtained with siCD92 control siRNA.


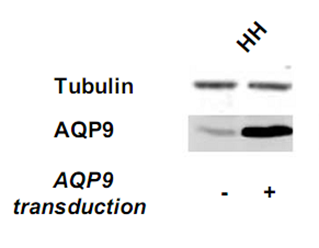


**Supplementary Figure 8. Human hepatocytes transduction with a lentiviral vector expressing hAQP9.** Hepatocytes were lysed in the wells 72 hours post-transduction. Cell lysates were then loaded onto 12% nonreducing SDS-PAGE. AQP9 expression was evaluated by immunoblotting as described in the materiel and methods section and compared to tubulin expression.


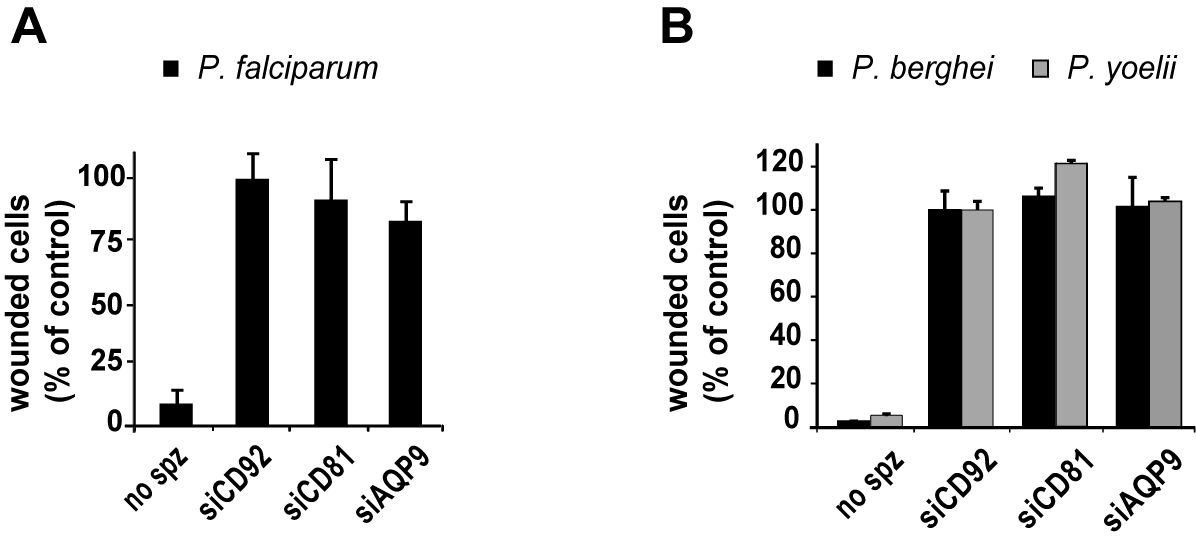


**Supplementary Figure 9. Cell traversal ability of *P. falciparum, P. berghei* and *P. yoelii* sporozoites in siRNA-treated liver cells.** HepaRG cells were transfected with sihCD92, sihCD81or sihAQP9^1^ three days post-transfection and incubated with *P. falciparum* **(A)**, *P. berghei* or *P. yoelii* **(B)** sporozoites in the presence of dextran-rhodamine. After 3 hours, cell traversal was assessed via FACS-mediated counting of dextran-positive wounded cells. “No spz” indicates the number of wounded cells after incubation with dextran rhodamine but without sporozoites. The results are expressed as the mean ± standard deviation.


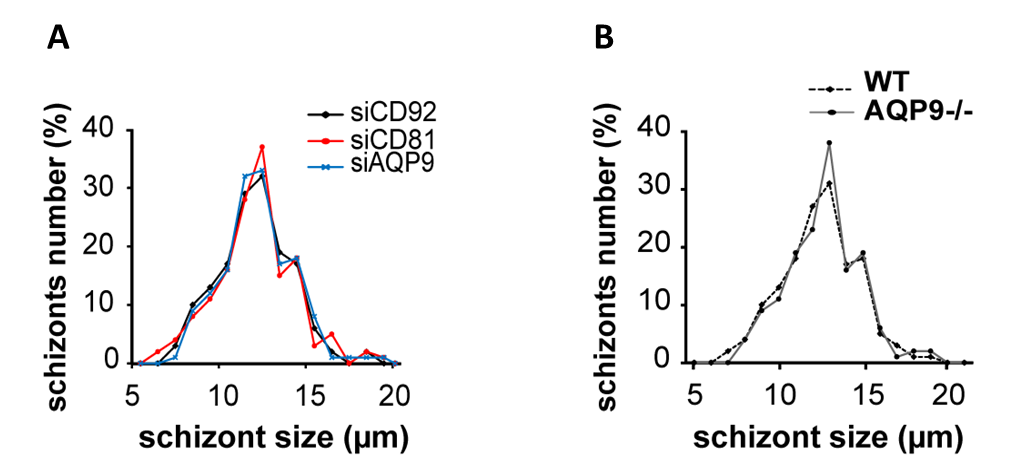


**Supplementary Figure 10.** **AQP9 is not involved in *Plasmodium* liver-stage development**. **(A)** SihAQP91-, sihCD81- and sihCD92-transfected human hepatocytes were infected with *P. falciparum* sporozoites and cultured for four days. **(B)** AQP9-knockout and wild type murine hepatocytes were infected with *P. berghei* sporozoites and cultured for two days. After labeling with the anti-HSP70 antibody, *P. falciparum* and *P. berghei* schizont diameters were measured under fluorescence microscopy. Schizont size distribution is reported for 100 schizonts.

**
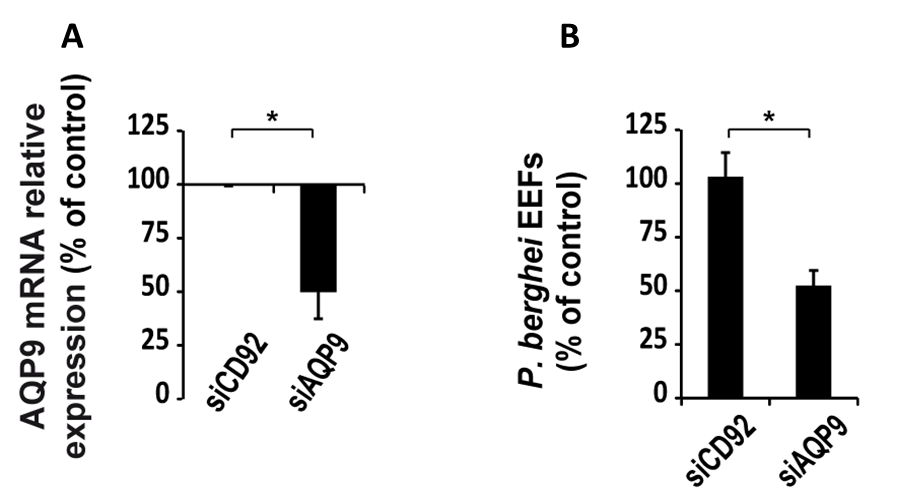
**

**Supplementary Figure 11. *P. berghei* infection of HepaRG cells, a human hepatoma cell line expressing AQP9, relies on AQP9 expression.** **(A)** RT-qPCR analysis of *AQP9* mRNA expression in sihCD92- and sihAQP9_1_-transfected HepaRG cells. Transcript levels were calculated after normalisation to TBP gene expression and are expressed relative to the *AQP9* mRNA level in sihCD92-transfected HepaRG cells. **(B) s**ihCD92- and sihAQP9_1_-transfected HepaRG cells were infected three days post-transfection with *P. berghei* sporozoites and cultured for two days until EEF quantification. EEF numbers are expressed as a percentage of those in control sihCD92-transfected cells. Analyses were carried out in triplicate wells, and the results are expressed as the mean ± standard deviation. The mean schizont numbers per well in controls of three experiments were 111, 85 and 91 *P. berghei* EEFs. *: *p*=0.05, determined by Mann-Whitney U test.
